# Supplementary material for: A Systematic Review to Evaluate Patient-Reported Outcome Measures (PROMs) for Metastatic Prostate Cancer According to the COnsensus-Based Standard for the Selection of Health Measurement INstruments (COSMIN) Methodology
Source: Cancers (Basel). 2022 Oct 19;14(20):5120. doi: 10.3390/cancers14205120 (PMC9600015; doi:10.3390/cancers14205120)
Supplement: Supplementary file 1 [file cancers-14-05120-s001.zip › Supplementary Table S1 The most frequently used PROMs in RCTs of men with mPCa were the.pdf]

Supplementary Table S1. PROMs used in RCTs evaluating mPCa patients.

**PROM**	**DOI of the publication**	**no. of RCT**
**FACT-P**	10.1016/S1470-2045(18)30898-2, 10.1016/S1470-2045(19)30620-5, 10.1016/S1470-2045(18)30456-X, 10.1016/j.eururo.2018.12.015, 10.1200/JCO.2017.75.3335, 10.1016/S1470-2045(17)30911-7, 10.1016/S1470-2045(15)70113-0, 10.1093/annonc/mdw065, 10.1093/annonc/mdx487, 10.1016/j.jval.2013.12.005, 10.1016/j.ejca.2017.09.035, 10.1016/j.eururo.2016.07.027, 10.1093/annonc/mdu510, 10.1016/j.eururo.2013.09.040, 10.1016/S1470-2045(20)30449-6.	18
**BPI-SF**	10.1016/S1470-2045(18)30898-2, 10.1016/j.eururo.2017.08.035, 10.1016/S1470-2045(20)30449-6, 10.1016/S1470-2045(17)30911-7, 10.1016/S1470-2045(15)70113-0, 10.1016/S1470-2045(14)70303-1, 10.1016/S1470-2045(13)70424-8, 10.1016/j.eururo.2014.10.001.	8
**EORTCQLQ-C30**	10.1002/pros.23317, 10.1016/j.annonc.2019.09.002, 10.1016/S1470-2045(20)30581-7, 10.1111/bju.13687, 10.1016/S1470-2045(17)30426-6, 10.1016/j.ejca.2020.08.019.	6
**BPI**	10.1007/s12149-018-1278-4, 10.1093/annonc/mdx487, 10.1016/j.ijrobp.2017.09.030.	4
**EORTCQLQ-PR25**	10.1111/bju.13687, 10.1186/s13014-019-1325-x, 10.1016/S1470-2045(18)30898-2.	3
**EQ-5D-5L**	10.1016/S1470-2045(19)30620-5, 10.1016/S1470-2045(20)30449-6, 10.1016/S1470-2045(17)30911-7.	3
**BFI**	10.1016/S1470-2045(19)30620-5, 10.1016/S1470-2045(17)30911-7.	3
