# Supplementary material for: A Systematic Review to Evaluate Patient-Reported Outcome Measures (PROMs) for Metastatic Prostate Cancer According to the COnsensus-Based Standard for the Selection of Health Measurement INstruments (COSMIN) Methodology
Source: Cancers (Basel). 2022 Oct 19;14(20):5120. doi: 10.3390/cancers14205120 (PMC9600015; doi:10.3390/cancers14205120)
Supplement: Supplementary file 1 [file cancers-14-05120-s001.zip › Supplementary Table S2 Summary Card of the FACT-P.pdf]

**Instrument**	**Functional Assessment of Cancer Therapy-Prostate (FACT-P)**
Acronym	FACT-P
Core Domain	Quality of Life
Area assessed<br>(Number of questions)	39 items<br>Four domains:<br>-Physical<br>-Social/family<br>-Emotional<br>-Functional well-being
Description	Developed to assess the quality of life of prostate cancer patients
Recall period	7 days
Scoring information	5-point Likert scale ranging from not at all to very much (0–4)
Estimated completion time	< 15 minutes
Mode of administration	Self-administer
Contact and copyright information	Copyright 1987-1997. Questionnaires and all related subscales, translations, and adaptations are owned and copyrighted by David Cella.
Licensing and equipment cost	Free for use. There is no fee required to use FACT-P.
Number of RCTs evaluating instrument	18
Highest COSMIN rating	**Clark et al., 2014**<br>- Internal consistency: Cronbach's α 0.78-0.83 (+);<br>COSMIN: High<br>- Reliability: ICC 0.70 (+);<br>COSMIN: Moderate<br>- Hypotheses testing: results in line with hypotheses (+);<br>COSMIN: High<br>- Responsiveness: clinically meaningful change of 6 to 10 (score range: 0–155)<br>COSMIN: High<br><br>**Robinson et al., 2013**<br>- Structural validity: exploratory analysis<br>COSMIN: Moderate<br>-Internal consistency: Cronbach's α 0.78-0.93 (+);<br>COSMIN: High<br>- Reliability: ICC 0.85-0-90 (+);<br>COSMIN: High<br>- Criterion validity: McNemar test: <16 (+);<br>COSMIN: Moderate

	- Hypotheses testing: results in line with hypotheses (+);<br>COSMIN: High

Supplementary Table S2: Summary Card of the FACT-P
