# Supplementary material for: A Systematic Review to Evaluate Patient-Reported Outcome Measures (PROMs) for Metastatic Prostate Cancer According to the COnsensus-Based Standard for the Selection of Health Measurement INstruments (COSMIN) Methodology
Source: Cancers (Basel). 2022 Oct 19;14(20):5120. doi: 10.3390/cancers14205120 (PMC9600015; doi:10.3390/cancers14205120)
Supplement: Supplementary file 1 [file cancers-14-05120-s001.zip › Supplementary Table S3 Summary card of the BPI.pdf]

Supplementary Table S3: Summary Card of the BPI

**Instrument**	**Brief Pain Inventory (BPI)**
Acronym	BPI
Core Domain	Pain
Area assessed<br>(Number of questions)	11 items:<br>4 items for the intensity of pain in different situations<br>7 items for measuring pain interference
Description	Developed to assess the severity and the impact of pain on daily functions
Recall period	24-hours
Scoring information	3 to 6-point Likert scale linearly transformed to 0-100 scale
Estimated completion time	< 10 minutes
Mode of administration	Self-administer
Contact and copyright information	Charles S. Cleeland, PhD, Pain Research Group - All right reserved
Licensing and equipment cost	Free for use.
Number of RCTs evaluating instrument	4
Highest COSMIN rating	**Robinson et al., 2013**<br>- Internal consistency: Cronbach's α 0.72-0.94 (+);<br>COSMIN: High<br>- Reliability: ICC 0.73-0-90; Kappa coeff. 0.56 (+);<br>COSMIN: High<br>- Criterion validity: McNemar test: >0 (+);<br>COSMIN: Moderate
