# Supplementary material for: A Systematic Review to Evaluate Patient-Reported Outcome Measures (PROMs) for Metastatic Prostate Cancer According to the COnsensus-Based Standard for the Selection of Health Measurement INstruments (COSMIN) Methodology
Source: Cancers (Basel). 2022 Oct 19;14(20):5120. doi: 10.3390/cancers14205120 (PMC9600015; doi:10.3390/cancers14205120)
Supplement: Supplementary file 1 [file cancers-14-05120-s001.zip › Supplementary Table S4 Summary card BPI-SF.pdf]

Supplementary Table S4: Summary Card of the BPI-SF

Instrument	Brief Pain Inventory - Short form (BPI-SF)
Acronym	BPI-SF
Core Domain	Pain
Area assessed<br>(Number of questions)	9 items to evaluate:<br><br>-The severity of a patient's pain<br><br>-The impact of the pain on patient's daily functioning
Description	Developed to assess the severity and the impact of pain on daily functions
Recall period	24-hours
Scoring information	11-point numerical rating scale (NRS) ranging from 0 to 10
Estimated completion time	< 10 minutes
Mode of administration	Self-administer
Contact and copyright information	BPI-SF © Copyright 1991 Charles S. Cleeland, PhD. Pain Research Group – Used by permission. All rights reserved.
Licensing and equipment cost	Free for use.
Number of RCTs evaluating instrument	8
Highest COSMIN rating	**Clark et al., 2014**<br>- Reliability: ICC 0.73-0.90<br>COSMIN: High<br>-Hypotheses testing: results in line with hypotheses (+);<br>COSMIN: High<br>- Responsiveness: r = −0.42; P < 0.006 and r = 0.57; P < 0.006 (+);<br>COSMIN: High<br><br>**Gater et al., 2011**<br>- Structural validity: no evidence (-)<br>COSMIN: Very Low
