# Supplementary material for: A Systematic Review to Evaluate Patient-Reported Outcome Measures (PROMs) for Metastatic Prostate Cancer According to the COnsensus-Based Standard for the Selection of Health Measurement INstruments (COSMIN) Methodology
Source: Cancers (Basel). 2022 Oct 19;14(20):5120. doi: 10.3390/cancers14205120 (PMC9600015; doi:10.3390/cancers14205120)
Supplement: Supplementary file 1 [file cancers-14-05120-s001.zip › Supplementary Table S5 Characteristics of the included studies.pdf]

Supplementary Table S5. Characteristics of included studies

PROM			Article information	Patient characteristics					Questionnaires	
**Instruments**	**Type**	**Content**	**Authors**	**Populations**	**N**	**Age Years Mean (SD)**	**Disease duration Years Mean (SD)**	**Disease severity Gleason Score, PSA, Jewet stage, EDSS/S&E/SA-SIP-30 Median (IQR)**	**Investigated**	**Language version**
**FACT-P**	Specific for PCa	39 items which assess physical, social/family, emotional and functional well-being domains.	Clark et al., 2014	43	mPCa	Not reported	Not reported	Not reported	FACT-P,<br>BPI-SF,<br>PPI	English
**FACT-P**	Specific for PCa	39 items which assess physical, social/family, emotional and functional well-being domains.	Robinson et al., 2013	76-131[a]	mPCa	67.7 (8.6)<br>68.4 (8.6)<br>66.7 (8.1)<br>66.9 (8.3)	Not reported	7.7(1.0)<br>7.4 (1.6)<br>7.3(1.6)<br>393.5 (641.3)<br>417.1 (691.1)<br>279.0 (799.6)<br>225.2 (412.7)	FACT-P,<br>BPI,<br>PPI	English
**BPI-SF**	Generic	9 items to evaluate the severity of a patient's pain and the impact of the pain on patient's daily functioning	Clark et al., 2014	43	mPCa	Not reported	Not reported	Not reported	BPI-SF,<br>FACT-P,<br>PPI	English
**BPI-SF**	Generic	9 items to evaluate the severity of a patient's pain and the impact of the pain on patient's daily functioning	Gater et al., 2011	17	mPCa	71.1 [53 - 86]	7.0<br>1.7	2-4 12%<br>5-7 29%<br>8-10 29%	BPI-SF,<br>PPI,<br>MPQ	English
**BPI**	Generic	11 items: 4 items to assess the intensity of pain in different situations and 7 items for measuring pain interference	Robinson et al., 2013	76-131[a]	mPCa	67.7 (8.6)<br>68.4 (8.6)<br>66.7 (8.1)<br>66.9 (8.3)	Not reported	7.7(1.0)<br>7.4 (1.6)<br>7.3(1.6)<br>393.5 (641.3)<br>417.1 (691.1)<br>279.0 (799.6)<br>225.2 (412.7)	BPI,<br>PPI,<br>FACT-P	Japanese

[a] Range of different (sub)sample

[b] Means years since prostate cancer diagnosis

[c] Means years since onset of bone metastases
